# 3,3′-Dinitro­bis­phenol A

**DOI:** 10.1107/S1600536811035458

**Published:** 2011-09-03

**Authors:** Sainath Babu, Chintan Pathak, Satvika Uppu, Conrad Jones, Frank R. Fronczek, Rao M. Uppu

**Affiliations:** a Department of Environmental Toxicology, Southern University and A&M College, Baton Rouge, Louisiana 70813, USA; b Department of Chemistry, Southern University and A&M College, Baton Rouge, Louisiana 70813, USA; c Department of Chemistry, Louisiana State University, Baton Rouge, Louisiana 70803, USA

## Abstract

The title compound [systematic name: 2,2′-dinitro-4,4′-(propane-2,2-di­yl)diphenol], C_15_H_14_N_2_O_6_, crystallizes with two mol­ecules in the asymmetric unit. Both have a *trans* conformation for their OH groups, and in each, the two aromatic rings are nearly orthogonal, with dihedral angles of 88.30 (3) and 89.62 (2)°. The nitro groups are nearly in the planes of their attached benzene rings, with C—C—N—O torsion angles in the range 1.21 (17)–4.06 (17)°, and they each accept an intra­molecular O—H⋯O hydrogen bond from their adjacent OH groups. One of the OH groups also forms a weak inter­molecular O—H⋯O hydrogen bond.

## Related literature

For background information on bis­phenol A and its uses and environmental effects, see: Hong-Mei & Nicell (2008 ▶); Lang *et al.* (2008 ▶); Masuda *et al.* (2005 ▶); Murrell (2006 ▶); Nakamura *et al.* (2011 ▶); Richter *et al.* (2007 ▶); Sakuyama *et al.* (2003 ▶); Toyoizumi *et al.* (2007 ▶); Vandenberg *et al.* (2009 ▶); Wang *et al.* (2007 ▶). For related structures, see: Bel’skii *et al.* (1983 ▶); Goldberg *et al.* (1991 ▶); Lim & Tanski (2007 ▶); Okada (1996 ▶); Wang *et al.* (1982 ▶). For graph-set analysis, see: Etter (1990 ▶).
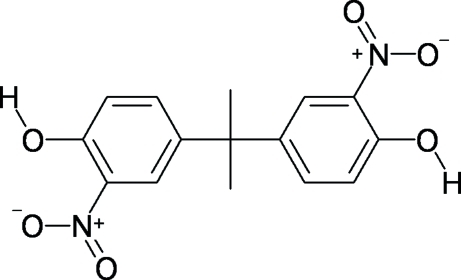


## Experimental

### 

#### Crystal data

C_15_H_14_N_2_O_6_
*M* *_r_* = 318.28
Triclinic, 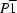

*a* = 8.3989 (5) Å
*b* = 12.5738 (7) Å
*c* = 15.3757 (9) Å
α = 66.967 (2)°
β = 76.565 (2)°
γ = 77.833 (2)°
*V* = 1440.34 (14) Å^3^
*Z* = 4
Cu *K*α radiation
μ = 0.98 mm^−1^
*T* = 90 K
0.30 × 0.24 × 0.15 mm

#### Data collection

Bruker Kappa APEXII CCD diffractometer
Absorption correction: multi-scan (*SADABS*; Sheldrick, 2004 ▶) *T*_min_ = 0.758, *T*_max_ = 0.867
17716 measured reflections
5276 independent reflections
5010 reflections with *I* > 2σ(*I*)
*R*_int_ = 0.028

#### Refinement

*R*[*F* ^2^ > 2σ(*F* ^2^)] = 0.034
*wR*(*F* ^2^) = 0.092
*S* = 1.04
5276 reflections
432 parameters
H atoms treated by a mixture of independent and constrained refinement
Δρ_max_ = 0.32 e Å^−3^
Δρ_min_ = −0.19 e Å^−3^

### 

Data collection: *APEX2* (Bruker, 2006 ▶); cell refinement: *SAINT* (Bruker, 2006 ▶); data reduction: *SAINT*; program(s) used to solve structure: *SHELXS97* (Sheldrick, 2008 ▶); program(s) used to refine structure: *SHELXL97* (Sheldrick, 2008 ▶); molecular graphics: *ORTEP-3 for Windows* (Farrugia, 1997 ▶); software used to prepare material for publication: *SHELXTL* (Sheldrick, 2008 ▶).

## Supplementary Material

Crystal structure: contains datablock(s) global, I. DOI: 10.1107/S1600536811035458/hb6392sup1.cif

Structure factors: contains datablock(s) I. DOI: 10.1107/S1600536811035458/hb6392Isup2.hkl

Supplementary material file. DOI: 10.1107/S1600536811035458/hb6392Isup3.cml

Additional supplementary materials:  crystallographic information; 3D view; checkCIF report

## Figures and Tables

**Table 1:** Hydrogen-bond geometry (Å, °)

*D*—H⋯*A*	*D*—H	H⋯*A*	*D*⋯*A*	*D*—H⋯*A*
O1—H1⋯O3	0.88 (2)	1.80 (2)	2.5947 (14)	149.6 (18)
O2—H2⋯O5	0.844 (19)	1.856 (18)	2.5955 (13)	145.3 (16)
O2—H2⋯O5^i^	0.844 (19)	2.380 (18)	2.9832 (13)	128.8 (14)
O7—H7⋯O9	0.91 (2)	1.72 (2)	2.5667 (17)	154 (2)
O8—H8⋯O11	0.85 (2)	1.81 (2)	2.5747 (14)	148.8 (19)

Symmetry code: (i) 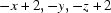.

**Fig. 1.**
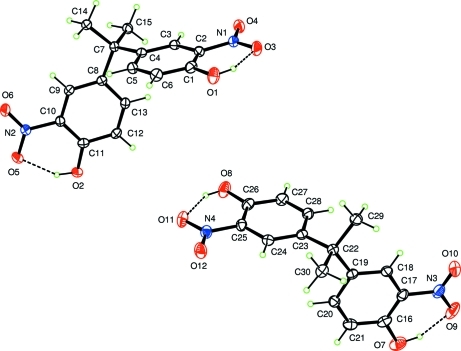
The molecular structure of (I) with ellipsoids at the 50% level, with H atoms having arbitrary radius.

**Fig. 2.**
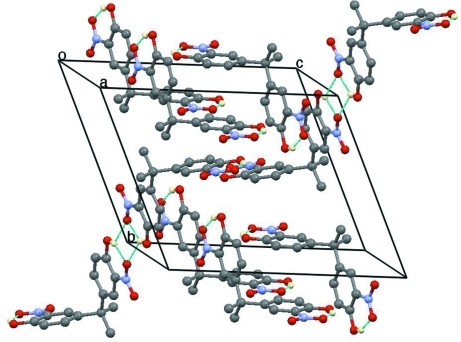
The unit cell, showing hydrogen bonding.

**Fig. 3.**
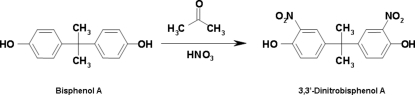
Nitration of bisphenol A by nitric acid/acetone mixtures at 0–5°C.

**Fig. 4.**
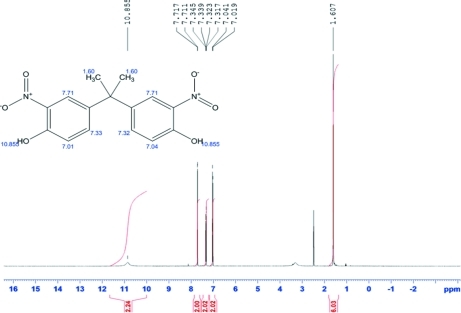
1H-NMR spectrum of 3,3'-dinitrobisphenol A.

**Fig. 5.**
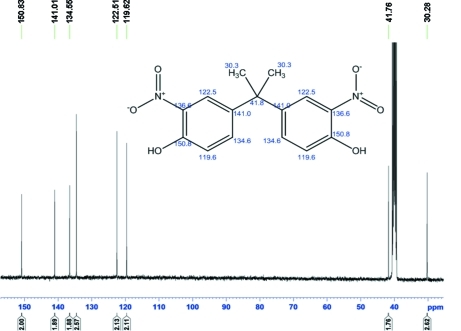
13C-NMR spectrum of 3,3'-dinitrobisphenol A.

**Fig. 6.**
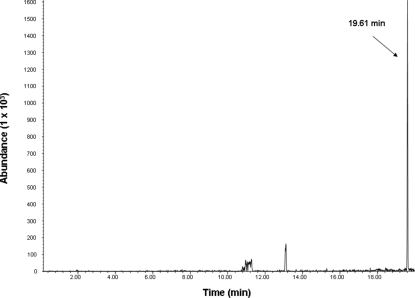
Ion chromatogram of the silylated product of 3,3'- dinitrobisphenol A.

**Fig. 7.**
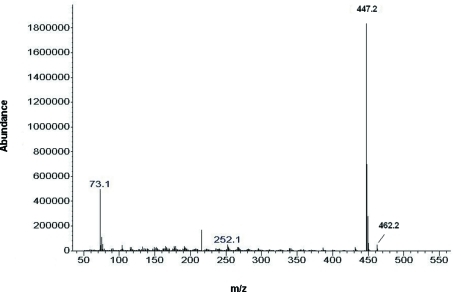
Electron ionization fragmentation of the silylated product of 3,3'-dintrobisphenol A.

**Fig. 8.**
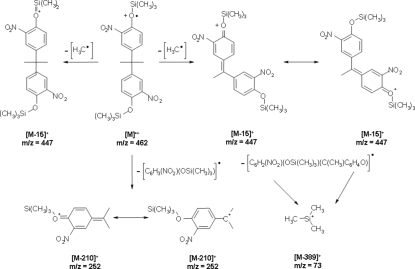
Proposed routes of fragmentation of the molecular ion of silylated 3,3'-dinitrobisphenol A.

## supplementary crystallographic information

### Comment

Bisphenol A (BPA), a persistent organic pollutant in urban environments, ranks among the top 2% of high volume chemicals produced in the United States (Vandenberg *et al.*, 2009). It is used in the making of printing materials, polycarbonate plastics and resins, all of which can become portals for human exposure. Studies using experimental animals have repeatedly shown that low level exposures to BPA can cause liver and brain damage, impaired insulin secretion, and a dysfunctional reproductive system (Richter *et al.*, 2007). A recent report by Lang and colleagues (Lang *et al.*, 2008), who analyzed the results of the first major epidemiologic study conducted by the National Health and Nutrition Examination Survey (NHANES) in 2003–2004, suggests wide-spread exposure to BPA in noninstitutionalized US populations where urinary levels of BPA are positively associated with higher incidence of cardiovascular diseases, diabetes, and abnormal concentrations of liver enzymes.

Bisphenol A can under go metabolic transformation by enzymes of cytochrome P450 system (Nakamura *et al.*, 2011), peroxidases (Hong-Mei & Nicell, 2008; Sakuyama *et al.*, 2003), and neutrophil and macrophage derived oxidants such as hypochlorous acid (Wang *et al.*, 2007) and peroxynitrite (B.Martin, S. Babu, C. Pathak & *R*. Uppu, unpublished work). It is likely that the various putative metabolites of BPA formed in these reactions can act as secondary toxins and relay, at least, in part, the toxic effects of BPA. It has been shown, for instance, that nitrated and chlorinated products of BPA, which could be formed *in vivo* in hypochlorous acid and peroxynitrite mediated oxidations, exhibit higher toxicity than BPA itself and often elaborate mutagenic and/or genotoxic effects (Masuda *et al.*, 2005; Toyoizumi *et al.*, 2007). To better understand the molecular targets for nitrated products of BPA and the likely disruption of endocrine function, in the present report we have synthesized 3,3'-dinitrobisphenol A, (I), using acetone/nitric acid mixtures and characterized the product by IR, NMR, and GC-MS following necessary purification and recrystallization from ethanol. The structural co-ordinates obtained from the crystallographic studies are presented here with the anticipation that they could be used in computational studies aimed at understanding the molecular docking and energetics of binding to possible targets such as the androgen and estrogen receptors and serum proteins.

The two independent molecules in the asymmetric unit are illustrated in Fig. 1. In both, the conformation is *trans*, placing the OH groups on the opposite sides of the molecule. The phenyl groups are twisted with respect to the central C(CH_3_)_2_ group such that they are nearly perpendicular. The dihedral angle formed by the two phenyl planes is 89.62 (2)° in the molecule containing C1 through C15 and 88.30 (3)° in the second molecule. The nitro groups lie near the planes of the phenyl groups and form intramolecular hydrogen bonds with the adjacent OH groups. The C1—C2—N1—O3 torsion angle is 4.06 (17)°, and analogous torsion angles involving the other nitro groups are 2.11 (17)° for N2, 3.73 (18)° for N3, and 1.21 (17)° for N4. The intramolecular hydrogen bonds have O···O distances in the range 2.5667 (17) - 2.5955 (13) Å. One OH group, O2 also has an intermolecular acceptor at a longer distance, O2···O5 (at 2 - *x*, -*y*, 2 - *z*), 2.9832 (13) Å, forming centrosymmetric *R*^2^_2_(4) rings (Etter, 1990), as shown in Fig. 2.

Similar intramolecular hydrogen bonds are found in the crystal structure of 2,2',6,6'-tetranitro-4,4-isopropylidenediphenol (Wang *et al.*, 1982), which has two nitro groups adjacent to each OH. As in the title structure, the hydrogen- bonded nitro group lies near the plane of the phenyl group, while the other nitro group twists 46.5 (3)° out of plane.

### Experimental

Chemicals and solvents used in the synthesis and recrystallization of 3,3'-dinitrobisphenol A were obtained as follows: BPA and DMSO-d6 from Sigma-Aldrich (St. Louis, MO); HNO3 (70% *w*/*v*; density: 1.42 g/ml) from Fisher Scientific (Fairlawn, NJ); acetone from Mallinckrodt (Phillipsburg, NJ); and TRI-SIL/TBT reagent (a special formulation of silylation mixture consisting of TMS-imidazole with bis-TMS-acetamide and trimethylchlorosilane) from Pierce (Rockford, IL). Water used was ultrapure with resistance 18.2 MO/cm.

Nitration of BPA was performed according to the method of Murrell (2006) with minor modifications (Fig. 3). Briefly, BPA (5.1 g; 22.4 mmol) was dissolved in 50 ml of acetone in a round-bottom flask and HNO_3_ (4.2 ml; 66.3 mmol) was added drop-wise over a period of 30 min with continuous mixing. Throughout the course of nitration, the temperature of the flask was maintained at 0–5°C using an ice-water bath. At the end of HNO_3_ addition, the reaction mixture was brought to room temperature over a period of 1 h, while the contents were continuously stirred and then quenched in cold water. The yellow/orange precipitate was filtered and washed with an ice cold mixture of acetone and water (3:1). The precipitate was dried for 24 h at 37°C and purified further by recrystallization from ethanol.

The nitro product of BPA was dissolved in DMSO-d6 and analyzed for ^1^H– and ^13^C-NMR spectra using a Bruker Avance II 400 MHz spectrometer. The ^1^H-NMR showed peaks with chemical shifts (in p.p.m.) at δ: 10.86 (s, 2H), 7.71 (d, J = 2.54 Hz, 2H), 7.32 (dd, J = 2.50 and 2.50 Hz, 2H), 7.01(d, J = 8.55 Hz, 2H), and 1.60 (s, 6H) (Fig. 4). ^13^C-NMR spectrum showed chemical shifts (in p.p.m.) at δ: 30.28, (–CH_3_); 41.76 (C7), 119.62(C1), 122.51(C4), 134.55 (C6), 136.57(C3), and 141.01(C5), 150.83 (C2) (Fig. 5). The chemicals shifts and assignments of C and H shown are consistent with the structure shown in Fig. 1. Following silylation using the TRI-SIL/TBT reagent, the nitroproduct of BPA was analyzed by GC-MS using an Agilent Technologies 7890 A gas chromatograph equipped with an Agilent Technologies 5975 C V L triple-axis MSD and a HP-5MS capillary column (length: 30 m; internal diameter: 0.25 mm; and film thickness: 0.25 µm). Helium was used as the carrier gas (total flow: 3 ml/min; split ratio: 1:50) with temperature programming as follows: 40 °C for 2 min (isothermal); 20 °C/min up to 150 °C (ramp 1), 150 °C for 3 min (isothermal); 20 °C/min up to 300 °C (ramp 2), and 300 °C for 2 min (isothermal) (total run time: 20 min; temperature of the inlet port: 270°C). Under these conditions, the silylated product of nitro-BPA resolved as a single peak with a retention time of 19.61 min (Fig. 6). The ion chromatogram of the product eluting at 19.61 min showed a molecular ion (*M*^+.^) at m/*z* 462 (2.14%; relative to the base peak) and other fragments at m/*z* values of 447 (100%; base peak; [*M*-15]^+^ or [M—CH_3_]^+^), 252 (2.64%; [*M*-210]^+^ or [M—C_9_H_12_NO_3_Si]^+^), and 73 (42.54%; [*M*-389]^+^ or [M—C_18_H_21_N_2_O_6_Si]^+^ (Fig. 7). The proposed routes of fragmentation of the molecular ion of silylated 3,3'-dinitrobisphenol A, giving various daughter ions, are given in Fig. 8.

Yellow needles of 3,3'-dinitrobisphenol A were obtained from ethanol.

### Refinement

H atoms on C were placed in idealized positions, with C—H distances 0.95 - 0.98 Å. A torsional parameter was refined for each methyl group. Hydroxy H atom positions were refined. *U*_iso_ for H were assigned as 1.2 times *U*_eq_ of the attached atoms (1.5 for methyl and OH).

### Crystal data

**Table:**

C_15_H_14_N_2_O_6_	*Z* = 4
*M**_r_* = 318.28	*F*(000) = 664
Triclinic, *P*1	*D*_x_ = 1.468 Mg m^−^^3^
Hall symbol: -P 1	Cu *K*α radiation, λ = 1.54178 Å
*a* = 8.3989 (5) Å	Cell parameters from 9978 reflections
*b* = 12.5738 (7) Å	θ = 3.8–69.4°
*c* = 15.3757 (9) Å	µ = 0.98 mm^−^^1^
α = 66.967 (2)°	*T* = 90 K
β = 76.565 (2)°	Needle fragment, yellow
γ = 77.833 (2)°	0.30 × 0.24 × 0.15 mm
*V* = 1440.34 (14) Å^3^	

### Data collection

**Table:**

Bruker Kappa APEXII CCD diffractometer	5276 independent reflections
Radiation source: fine-focus sealed tube	5010 reflections with *I* > 2σ(*I*)
graphite	*R*_int_ = 0.028
φ and ω scans	θ_max_ = 69.8°, θ_min_ = 3.2°
Absorption correction: multi-scan (*SADABS*; Sheldrick, 2004)	*h* = −8→10
*T*_min_ = 0.758, *T*_max_ = 0.867	*k* = −15→15
17716 measured reflections	*l* = −16→18

### Refinement

**Table:**

Refinement on *F*^2^	Secondary atom site location: difference Fourier map
Least-squares matrix: full	Hydrogen site location: inferred from neighbouring sites
*R*[*F*^2^ > 2σ(*F*^2^)] = 0.034	H atoms treated by a mixture of independent and constrained refinement
*wR*(*F*^2^) = 0.092	*w* = 1/[σ^2^(*F*_o_^2^) + (0.0456*P*)^2^ + 0.5723*P*] where *P* = (*F*_o_^2^ + 2*F*_c_^2^)/3
*S* = 1.04	(Δ/σ)_max_ < 0.001
5276 reflections	Δρ_max_ = 0.32 e Å^−^^3^
432 parameters	Δρ_min_ = −0.19 e Å^−^^3^
0 restraints	Extinction correction: *SHELXL97* (Sheldrick, 2008), Fc^*^=kFc[1+0.001xFc^2^λ^3^/sin(2θ)]^-1/4^
Primary atom site location: structure-invariant direct methods	Extinction coefficient: 0.0019 (2)

### Special details

**Table:**

Geometry. All e.s.d.'s (except the e.s.d. in the dihedral angle between two l.s. planes) are estimated using the full covariance matrix. The cell e.s.d.'s are taken into account individually in the estimation of e.s.d.'s in distances, angles and torsion angles; correlations between e.s.d.'s in cell parameters are only used when they are defined by crystal symmetry. An approximate (isotropic) treatment of cell e.s.d.'s is used for estimating e.s.d.'s involving l.s. planes.
Refinement. Refinement of *F*^2^ against ALL reflections. The weighted *R*-factor *wR* and goodness of fit *S* are based on *F*^2^, conventional *R*-factors *R* are based on *F*, with *F* set to zero for negative *F*^2^. The threshold expression of *F*^2^ > σ(*F*^2^) is used only for calculating *R*-factors(gt) *etc*. and is not relevant to the choice of reflections for refinement. *R*-factors based on *F*^2^ are statistically about twice as large as those based on *F*, and *R*- factors based on ALL data will be even larger.

### Fractional atomic coordinates and isotropic or equivalent isotropic displacement parameters (Å^2^)

**Table:**

	*x*	*y*	*z*	*U*_iso_*/*U*_eq_	
O1	0.22904 (13)	0.58866 (9)	0.48806 (7)	0.0294 (2)	
H1	0.123 (3)	0.5893 (17)	0.4943 (14)	0.044*	
O2	0.73621 (11)	0.03318 (7)	0.95361 (6)	0.02063 (19)	
H2	0.829 (2)	0.0338 (15)	0.9658 (12)	0.031*	
O3	−0.07766 (12)	0.57717 (8)	0.56906 (7)	0.0292 (2)	
O4	−0.15232 (11)	0.55090 (9)	0.71963 (7)	0.0287 (2)	
O5	0.97246 (11)	0.12665 (8)	0.96739 (7)	0.0272 (2)	
O6	0.93932 (12)	0.30789 (8)	0.95141 (9)	0.0350 (3)	
N1	−0.04492 (13)	0.55927 (9)	0.64899 (8)	0.0224 (2)	
N2	0.88736 (13)	0.22431 (9)	0.95078 (8)	0.0212 (2)	
C1	0.25048 (16)	0.56709 (11)	0.57760 (9)	0.0216 (3)	
C2	0.12602 (15)	0.54936 (10)	0.65832 (9)	0.0194 (3)	
C3	0.16117 (15)	0.52384 (10)	0.74984 (9)	0.0176 (2)	
H3	0.0743	0.5110	0.8034	0.021*	
C4	0.32013 (14)	0.51728 (10)	0.76286 (8)	0.0163 (2)	
C5	0.44549 (15)	0.53631 (10)	0.68122 (9)	0.0197 (3)	
H5	0.5562	0.5318	0.6886	0.024*	
C6	0.41164 (16)	0.56111 (11)	0.59161 (9)	0.0225 (3)	
H6	0.4988	0.5744	0.5383	0.027*	
C7	0.36794 (14)	0.48809 (10)	0.86086 (8)	0.0162 (2)	
C8	0.47801 (14)	0.36988 (10)	0.88453 (8)	0.0153 (2)	
C9	0.63366 (14)	0.35074 (10)	0.90618 (8)	0.0166 (2)	
H9	0.6805	0.4137	0.9054	0.020*	
C10	0.72413 (14)	0.23857 (10)	0.92944 (8)	0.0169 (2)	
C11	0.66033 (15)	0.14318 (10)	0.93106 (8)	0.0167 (2)	
C12	0.50322 (15)	0.16441 (10)	0.90675 (8)	0.0177 (2)	
H12	0.4569	0.1023	0.9056	0.021*	
C13	0.41528 (14)	0.27400 (10)	0.88453 (8)	0.0172 (2)	
H13	0.3086	0.2859	0.8686	0.021*	
C14	0.45545 (14)	0.58693 (10)	0.85565 (9)	0.0178 (2)	
H14A	0.5557	0.5933	0.8072	0.027*	
H14B	0.4851	0.5697	0.9182	0.027*	
H14C	0.3812	0.6607	0.8382	0.027*	
C15	0.21674 (14)	0.47754 (11)	0.94100 (9)	0.0187 (2)	
H15A	0.1425	0.5515	0.9260	0.028*	
H15B	0.2531	0.4593	1.0020	0.028*	
H15C	0.1582	0.4151	0.9461	0.028*	
O7	0.02131 (15)	−0.30550 (9)	0.30590 (8)	0.0365 (3)	
H7	−0.080 (3)	−0.2877 (18)	0.2880 (15)	0.055*	
O8	0.33303 (13)	0.14153 (10)	0.57674 (7)	0.0320 (2)	
H8	0.435 (3)	0.1460 (17)	0.5692 (14)	0.048*	
O9	−0.24524 (14)	−0.19241 (10)	0.24194 (8)	0.0392 (3)	
O10	−0.28330 (13)	−0.00440 (11)	0.18519 (9)	0.0416 (3)	
O11	0.63606 (12)	0.15267 (9)	0.49364 (7)	0.0294 (2)	
O12	0.71117 (11)	0.15854 (9)	0.34782 (7)	0.0308 (2)	
N3	−0.20026 (14)	−0.09626 (11)	0.22333 (9)	0.0295 (3)	
N4	0.60419 (13)	0.15289 (9)	0.41816 (8)	0.0229 (2)	
C16	0.05818 (17)	−0.19784 (11)	0.28560 (9)	0.0243 (3)	
C17	−0.04478 (15)	−0.09397 (12)	0.24732 (9)	0.0221 (3)	
C18	−0.00188 (15)	0.01496 (11)	0.23027 (8)	0.0194 (3)	
H18	−0.0754	0.0838	0.2053	0.023*	
C19	0.14606 (14)	0.02296 (10)	0.24948 (8)	0.0169 (2)	
C20	0.25088 (15)	−0.08150 (11)	0.28611 (8)	0.0200 (3)	
H20	0.3541	−0.0778	0.2994	0.024*	
C21	0.20879 (17)	−0.18877 (11)	0.30335 (9)	0.0238 (3)	
H21	0.2833	−0.2573	0.3276	0.029*	
C22	0.20487 (14)	0.13939 (10)	0.22818 (8)	0.0179 (2)	
C23	0.24781 (15)	0.13806 (10)	0.32030 (8)	0.0171 (2)	
C24	0.40497 (15)	0.14409 (10)	0.32903 (9)	0.0180 (2)	
H24	0.4928	0.1474	0.2768	0.022*	
C25	0.43654 (15)	0.14534 (10)	0.41427 (9)	0.0194 (3)	
C26	0.31108 (16)	0.14011 (11)	0.49325 (9)	0.0225 (3)	
C27	0.15235 (16)	0.13151 (11)	0.48457 (9)	0.0234 (3)	
H27	0.0646	0.1264	0.5371	0.028*	
C28	0.12238 (15)	0.13041 (11)	0.40085 (9)	0.0203 (3)	
H28	0.0136	0.1243	0.3970	0.024*	
C29	0.07223 (16)	0.24398 (11)	0.19345 (9)	0.0223 (3)	
H29A	−0.0281	0.2348	0.2419	0.033*	
H29B	0.0469	0.2477	0.1332	0.033*	
H29C	0.1133	0.3161	0.1832	0.033*	
C30	0.35372 (16)	0.15349 (11)	0.14636 (9)	0.0216 (3)	
H30A	0.3934	0.2279	0.1305	0.032*	
H30B	0.3203	0.1523	0.0898	0.032*	
H30C	0.4423	0.0893	0.1666	0.032*	

### Atomic displacement parameters (Å^2^)

**Table:**

	*U*^11^	*U*^22^	*U*^33^	*U*^12^	*U*^13^	*U*^23^
O1	0.0321 (5)	0.0389 (6)	0.0185 (5)	−0.0099 (4)	−0.0084 (4)	−0.0066 (4)
O2	0.0209 (4)	0.0159 (4)	0.0250 (5)	−0.0003 (3)	−0.0054 (4)	−0.0075 (3)
O3	0.0303 (5)	0.0322 (5)	0.0299 (5)	−0.0016 (4)	−0.0171 (4)	−0.0107 (4)
O4	0.0172 (4)	0.0393 (6)	0.0358 (5)	−0.0012 (4)	−0.0053 (4)	−0.0207 (4)
O5	0.0218 (5)	0.0205 (4)	0.0398 (5)	0.0046 (4)	−0.0127 (4)	−0.0110 (4)
O6	0.0249 (5)	0.0259 (5)	0.0648 (7)	0.0000 (4)	−0.0213 (5)	−0.0216 (5)
N1	0.0219 (5)	0.0199 (5)	0.0285 (6)	−0.0001 (4)	−0.0103 (5)	−0.0100 (4)
N2	0.0191 (5)	0.0199 (5)	0.0266 (5)	0.0000 (4)	−0.0077 (4)	−0.0097 (4)
C1	0.0280 (6)	0.0189 (6)	0.0184 (6)	−0.0035 (5)	−0.0071 (5)	−0.0053 (5)
C2	0.0185 (6)	0.0163 (6)	0.0248 (6)	−0.0004 (4)	−0.0078 (5)	−0.0073 (5)
C3	0.0175 (6)	0.0153 (5)	0.0204 (6)	−0.0010 (4)	−0.0031 (5)	−0.0075 (5)
C4	0.0172 (5)	0.0138 (5)	0.0182 (6)	−0.0009 (4)	−0.0042 (4)	−0.0058 (4)
C5	0.0171 (6)	0.0206 (6)	0.0215 (6)	−0.0026 (5)	−0.0034 (5)	−0.0077 (5)
C6	0.0226 (6)	0.0253 (6)	0.0188 (6)	−0.0063 (5)	0.0001 (5)	−0.0077 (5)
C7	0.0148 (5)	0.0164 (6)	0.0179 (6)	−0.0019 (4)	−0.0036 (4)	−0.0064 (4)
C8	0.0167 (5)	0.0168 (6)	0.0120 (5)	−0.0026 (4)	−0.0011 (4)	−0.0053 (4)
C9	0.0179 (6)	0.0167 (6)	0.0168 (5)	−0.0038 (4)	−0.0027 (4)	−0.0072 (4)
C10	0.0150 (5)	0.0201 (6)	0.0163 (5)	−0.0024 (4)	−0.0030 (4)	−0.0071 (5)
C11	0.0203 (6)	0.0159 (6)	0.0123 (5)	−0.0023 (4)	0.0000 (4)	−0.0049 (4)
C12	0.0203 (6)	0.0179 (6)	0.0169 (6)	−0.0070 (4)	−0.0006 (4)	−0.0075 (5)
C13	0.0157 (5)	0.0207 (6)	0.0163 (5)	−0.0041 (4)	−0.0025 (4)	−0.0070 (5)
C14	0.0183 (6)	0.0162 (6)	0.0204 (6)	−0.0021 (4)	−0.0050 (5)	−0.0071 (5)
C15	0.0161 (5)	0.0220 (6)	0.0189 (6)	−0.0020 (4)	−0.0021 (5)	−0.0092 (5)
O7	0.0443 (6)	0.0244 (5)	0.0442 (6)	−0.0143 (5)	0.0001 (5)	−0.0154 (5)
O8	0.0334 (5)	0.0488 (6)	0.0207 (5)	−0.0137 (5)	−0.0033 (4)	−0.0161 (4)
O9	0.0400 (6)	0.0526 (7)	0.0410 (6)	−0.0249 (5)	0.0004 (5)	−0.0283 (5)
O10	0.0264 (5)	0.0546 (7)	0.0569 (7)	0.0005 (5)	−0.0169 (5)	−0.0316 (6)
O11	0.0324 (5)	0.0335 (5)	0.0261 (5)	−0.0102 (4)	−0.0129 (4)	−0.0075 (4)
O12	0.0200 (5)	0.0425 (6)	0.0307 (5)	−0.0095 (4)	−0.0009 (4)	−0.0132 (4)
N3	0.0243 (6)	0.0443 (7)	0.0306 (6)	−0.0096 (5)	−0.0001 (5)	−0.0246 (6)
N4	0.0236 (5)	0.0203 (5)	0.0247 (6)	−0.0057 (4)	−0.0068 (5)	−0.0051 (4)
C16	0.0325 (7)	0.0219 (6)	0.0199 (6)	−0.0096 (5)	0.0034 (5)	−0.0105 (5)
C17	0.0209 (6)	0.0308 (7)	0.0197 (6)	−0.0074 (5)	0.0009 (5)	−0.0149 (5)
C18	0.0201 (6)	0.0225 (6)	0.0164 (6)	−0.0006 (5)	−0.0021 (4)	−0.0096 (5)
C19	0.0193 (6)	0.0188 (6)	0.0128 (5)	−0.0027 (5)	−0.0006 (4)	−0.0070 (4)
C20	0.0206 (6)	0.0216 (6)	0.0173 (6)	−0.0019 (5)	−0.0039 (5)	−0.0066 (5)
C21	0.0293 (7)	0.0186 (6)	0.0193 (6)	−0.0001 (5)	−0.0023 (5)	−0.0050 (5)
C22	0.0190 (6)	0.0173 (6)	0.0172 (6)	−0.0027 (5)	−0.0027 (5)	−0.0060 (5)
C23	0.0197 (6)	0.0137 (5)	0.0175 (6)	−0.0027 (4)	−0.0028 (5)	−0.0053 (4)
C24	0.0188 (6)	0.0156 (5)	0.0174 (6)	−0.0035 (4)	−0.0007 (4)	−0.0044 (4)
C25	0.0196 (6)	0.0172 (6)	0.0212 (6)	−0.0051 (4)	−0.0046 (5)	−0.0047 (5)
C26	0.0288 (7)	0.0224 (6)	0.0178 (6)	−0.0060 (5)	−0.0037 (5)	−0.0078 (5)
C27	0.0229 (6)	0.0276 (7)	0.0200 (6)	−0.0071 (5)	0.0027 (5)	−0.0104 (5)
C28	0.0181 (6)	0.0221 (6)	0.0216 (6)	−0.0045 (5)	−0.0016 (5)	−0.0088 (5)
C29	0.0257 (6)	0.0176 (6)	0.0230 (6)	−0.0006 (5)	−0.0070 (5)	−0.0060 (5)
C30	0.0240 (6)	0.0235 (6)	0.0171 (6)	−0.0072 (5)	−0.0002 (5)	−0.0068 (5)

### Geometric parameters (Å, °)

**Table:**

O1—C1	1.3431 (15)	O7—C16	1.3516 (16)
O1—H1	0.88 (2)	O7—H7	0.91 (2)
O2—C11	1.3398 (14)	O8—C26	1.3460 (15)
O2—H2	0.844 (19)	O8—H8	0.85 (2)
O3—N1	1.2456 (14)	O9—N3	1.2481 (16)
O4—N1	1.2246 (15)	O10—N3	1.2185 (17)
O5—N2	1.2464 (14)	O11—N4	1.2490 (14)
O6—N2	1.2240 (14)	O12—N4	1.2237 (15)
N1—C2	1.4504 (16)	N3—C17	1.4457 (17)
N2—C10	1.4418 (15)	N4—C25	1.4475 (16)
C1—C2	1.4005 (18)	C16—C21	1.3903 (19)
C1—C6	1.4014 (18)	C16—C17	1.3997 (19)
C2—C3	1.4037 (17)	C17—C18	1.3991 (18)
C3—C4	1.3763 (17)	C18—C19	1.3739 (17)
C3—H3	0.9500	C18—H18	0.9500
C4—C5	1.4138 (17)	C19—C20	1.4077 (17)
C4—C7	1.5339 (16)	C19—C22	1.5326 (16)
C5—C6	1.3729 (18)	C20—C21	1.3755 (18)
C5—H5	0.9500	C20—H20	0.9500
C6—H6	0.9500	C21—H21	0.9500
C7—C8	1.5331 (16)	C22—C23	1.5340 (16)
C7—C15	1.5377 (16)	C22—C29	1.5378 (16)
C7—C14	1.5398 (15)	C22—C30	1.5379 (16)
C8—C9	1.3729 (17)	C23—C24	1.3779 (17)
C8—C13	1.4137 (16)	C23—C28	1.4128 (17)
C9—C10	1.4051 (17)	C24—C25	1.4028 (17)
C9—H9	0.9500	C24—H24	0.9500
C10—C11	1.4028 (17)	C25—C26	1.4006 (18)
C11—C12	1.3981 (17)	C26—C27	1.4004 (18)
C12—C13	1.3715 (17)	C27—C28	1.3736 (18)
C12—H12	0.9500	C27—H27	0.9500
C13—H13	0.9500	C28—H28	0.9500
C14—H14A	0.9800	C29—H29A	0.9800
C14—H14B	0.9800	C29—H29B	0.9800
C14—H14C	0.9800	C29—H29C	0.9800
C15—H15A	0.9800	C30—H30A	0.9800
C15—H15B	0.9800	C30—H30B	0.9800
C15—H15C	0.9800	C30—H30C	0.9800
			
C1—O1—H1	103.2 (13)	C16—O7—H7	100.9 (14)
C11—O2—H2	106.1 (12)	C26—O8—H8	104.9 (14)
O4—N1—O3	122.20 (10)	O10—N3—O9	121.88 (12)
O4—N1—C2	119.00 (10)	O10—N3—C17	119.13 (12)
O3—N1—C2	118.80 (11)	O9—N3—C17	118.99 (12)
O6—N2—O5	121.75 (10)	O12—N4—O11	121.70 (11)
O6—N2—C10	119.57 (10)	O12—N4—C25	119.28 (10)
O5—N2—C10	118.68 (10)	O11—N4—C25	119.02 (10)
O1—C1—C2	125.71 (12)	O7—C16—C21	118.19 (12)
O1—C1—C6	117.15 (11)	O7—C16—C17	124.50 (13)
C2—C1—C6	117.14 (11)	C21—C16—C17	117.31 (11)
C1—C2—C3	121.63 (11)	C18—C17—C16	121.91 (12)
C1—C2—N1	120.44 (11)	C18—C17—N3	117.65 (12)
C3—C2—N1	117.92 (11)	C16—C17—N3	120.45 (12)
C4—C3—C2	120.71 (11)	C19—C18—C17	120.35 (11)
C4—C3—H3	119.6	C19—C18—H18	119.8
C2—C3—H3	119.6	C17—C18—H18	119.8
C3—C4—C5	117.64 (11)	C18—C19—C20	117.60 (11)
C3—C4—C7	123.54 (10)	C18—C19—C22	123.22 (11)
C5—C4—C7	118.81 (10)	C20—C19—C22	119.09 (10)
C6—C5—C4	121.82 (11)	C21—C20—C19	122.20 (12)
C6—C5—H5	119.1	C21—C20—H20	118.9
C4—C5—H5	119.1	C19—C20—H20	118.9
C5—C6—C1	121.04 (11)	C20—C21—C16	120.59 (12)
C5—C6—H6	119.5	C20—C21—H21	119.7
C1—C6—H6	119.5	C16—C21—H21	119.7
C8—C7—C4	107.24 (9)	C19—C22—C23	108.55 (9)
C8—C7—C15	108.16 (9)	C19—C22—C29	112.24 (10)
C4—C7—C15	112.37 (9)	C23—C22—C29	108.67 (9)
C8—C7—C14	112.85 (9)	C19—C22—C30	107.55 (9)
C4—C7—C14	108.79 (9)	C23—C22—C30	112.75 (10)
C15—C7—C14	107.52 (9)	C29—C22—C30	107.13 (10)
C9—C8—C13	117.69 (10)	C24—C23—C28	117.43 (11)
C9—C8—C7	124.27 (10)	C24—C23—C22	123.05 (10)
C13—C8—C7	118.04 (10)	C28—C23—C22	119.52 (10)
C8—C9—C10	120.35 (11)	C23—C24—C25	120.66 (11)
C8—C9—H9	119.8	C23—C24—H24	119.7
C10—C9—H9	119.8	C25—C24—H24	119.7
C11—C10—C9	121.81 (11)	C26—C25—C24	121.68 (11)
C11—C10—N2	120.50 (10)	C26—C25—N4	120.59 (11)
C9—C10—N2	117.69 (10)	C24—C25—N4	117.73 (11)
O2—C11—C12	116.73 (10)	O8—C26—C27	118.06 (11)
O2—C11—C10	126.02 (11)	O8—C26—C25	124.59 (12)
C12—C11—C10	117.25 (11)	C27—C26—C25	117.35 (11)
C13—C12—C11	120.63 (11)	C28—C27—C26	120.63 (11)
C13—C12—H12	119.7	C28—C27—H27	119.7
C11—C12—H12	119.7	C26—C27—H27	119.7
C12—C13—C8	122.25 (11)	C27—C28—C23	122.24 (11)
C12—C13—H13	118.9	C27—C28—H28	118.9
C8—C13—H13	118.9	C23—C28—H28	118.9
C7—C14—H14A	109.5	C22—C29—H29A	109.5
C7—C14—H14B	109.5	C22—C29—H29B	109.5
H14A—C14—H14B	109.5	H29A—C29—H29B	109.5
C7—C14—H14C	109.5	C22—C29—H29C	109.5
H14A—C14—H14C	109.5	H29A—C29—H29C	109.5
H14B—C14—H14C	109.5	H29B—C29—H29C	109.5
C7—C15—H15A	109.5	C22—C30—H30A	109.5
C7—C15—H15B	109.5	C22—C30—H30B	109.5
H15A—C15—H15B	109.5	H30A—C30—H30B	109.5
C7—C15—H15C	109.5	C22—C30—H30C	109.5
H15A—C15—H15C	109.5	H30A—C30—H30C	109.5
H15B—C15—H15C	109.5	H30B—C30—H30C	109.5
			
O1—C1—C2—C3	178.07 (11)	O7—C16—C17—C18	178.18 (11)
C6—C1—C2—C3	−1.52 (18)	C21—C16—C17—C18	−2.11 (18)
O1—C1—C2—N1	−3.15 (19)	O7—C16—C17—N3	−2.24 (19)
C6—C1—C2—N1	177.26 (11)	C21—C16—C17—N3	177.47 (11)
O4—N1—C2—C1	−175.24 (11)	O10—N3—C17—C18	3.63 (17)
O3—N1—C2—C1	4.06 (17)	O9—N3—C17—C18	−176.67 (11)
O4—N1—C2—C3	3.58 (16)	O10—N3—C17—C16	−175.96 (12)
O3—N1—C2—C3	−177.12 (10)	O9—N3—C17—C16	3.73 (18)
C1—C2—C3—C4	1.06 (18)	C16—C17—C18—C19	1.12 (18)
N1—C2—C3—C4	−177.74 (10)	N3—C17—C18—C19	−178.47 (10)
C2—C3—C4—C5	−0.42 (17)	C17—C18—C19—C20	0.22 (17)
C2—C3—C4—C7	−179.08 (10)	C17—C18—C19—C22	177.00 (10)
C3—C4—C5—C6	0.32 (17)	C18—C19—C20—C21	−0.52 (18)
C7—C4—C5—C6	179.05 (11)	C22—C19—C20—C21	−177.43 (11)
C4—C5—C6—C1	−0.85 (19)	C19—C20—C21—C16	−0.53 (19)
O1—C1—C6—C5	−178.22 (11)	O7—C16—C21—C20	−178.48 (11)
C2—C1—C6—C5	1.41 (18)	C17—C16—C21—C20	1.79 (18)
C3—C4—C7—C8	113.79 (12)	C18—C19—C22—C23	126.24 (11)
C5—C4—C7—C8	−64.86 (13)	C20—C19—C22—C23	−57.03 (13)
C3—C4—C7—C15	−4.93 (15)	C18—C19—C22—C29	6.11 (16)
C5—C4—C7—C15	176.43 (10)	C20—C19—C22—C29	−177.16 (10)
C3—C4—C7—C14	−123.86 (12)	C18—C19—C22—C30	−111.48 (12)
C5—C4—C7—C14	57.49 (13)	C20—C19—C22—C30	65.25 (13)
C4—C7—C8—C9	124.49 (11)	C19—C22—C23—C24	116.54 (12)
C15—C7—C8—C9	−114.11 (12)	C29—C22—C23—C24	−121.12 (12)
C14—C7—C8—C9	4.71 (16)	C30—C22—C23—C24	−2.51 (16)
C4—C7—C8—C13	−55.80 (13)	C19—C22—C23—C28	−63.49 (13)
C15—C7—C8—C13	65.61 (13)	C29—C22—C23—C28	58.84 (14)
C14—C7—C8—C13	−175.57 (10)	C30—C22—C23—C28	177.46 (10)
C13—C8—C9—C10	−1.38 (16)	C28—C23—C24—C25	−1.49 (17)
C7—C8—C9—C10	178.33 (10)	C22—C23—C24—C25	178.47 (10)
C8—C9—C10—C11	0.28 (17)	C23—C24—C25—C26	0.25 (18)
C8—C9—C10—N2	179.55 (10)	C23—C24—C25—N4	−179.43 (10)
O6—N2—C10—C11	−178.15 (11)	O12—N4—C25—C26	−179.27 (11)
O5—N2—C10—C11	2.44 (17)	O11—N4—C25—C26	1.21 (17)
O6—N2—C10—C9	2.57 (17)	O12—N4—C25—C24	0.42 (17)
O5—N2—C10—C9	−176.84 (11)	O11—N4—C25—C24	−179.11 (10)
C9—C10—C11—O2	−179.00 (11)	C24—C25—C26—O8	−179.66 (12)
N2—C10—C11—O2	1.75 (18)	N4—C25—C26—O8	0.01 (19)
C9—C10—C11—C12	1.15 (17)	C24—C25—C26—C27	1.07 (18)
N2—C10—C11—C12	−178.10 (10)	N4—C25—C26—C27	−179.26 (11)
O2—C11—C12—C13	178.70 (10)	O8—C26—C27—C28	179.59 (12)
C10—C11—C12—C13	−1.43 (17)	C25—C26—C27—C28	−1.10 (19)
C11—C12—C13—C8	0.33 (18)	C26—C27—C28—C23	−0.17 (19)
C9—C8—C13—C12	1.11 (17)	C24—C23—C28—C27	1.48 (18)
C7—C8—C13—C12	−178.63 (10)	C22—C23—C28—C27	−178.49 (11)

### Hydrogen-bond geometry (Å, °)

**Table:**

*D*—H···*A*	*D*—H	H···*A*	*D*···*A*	*D*—H···*A*
O1—H1···O3	0.88 (2)	1.80 (2)	2.5947 (14)	149.6 (18)
O2—H2···O5	0.844 (19)	1.856 (18)	2.5955 (13)	145.3 (16)
O2—H2···O5^i^	0.844 (19)	2.380 (18)	2.9832 (13)	128.8 (14)
O7—H7···O9	0.91 (2)	1.72 (2)	2.5667 (17)	154 (2)
O8—H8···O11	0.85 (2)	1.81 (2)	2.5747 (14)	148.8 (19)

Symmetry codes: (i) −*x*+2, −*y*, −*z*+2.
